# Supplementary material for: Assessment of the environmental impacts of conflict-driven Internally Displaced Persons: A sentinel-2 satellite based analysis of land use/cover changes in the Kas locality, Darfur, Sudan
Source: PLoS One. 2024 May 30;19(5):e0304034. doi: 10.1371/journal.pone.0304034 (PMC11139307; doi:10.1371/journal.pone.0304034)
Supplement: Attachment — Submitted filename: PONE-D-24-01879_reviewer_reviewed.pdf [file pone.0304034.s004.pdf]

PLOS ONE

Assessment of the environmental impacts of conflict-driven Internally Displaced Persons: A sentinel-2 satellite based analysis of vegetation cover changes in the Kas locality, Darfur, Sudan

--Manuscript Draft--

**Manuscript Number:**	PONE-D-24-01879
**Article Type:**	Research Article
**Full Title:**	Assessment of the environmental impacts of conflict-driven Internally Displaced Persons: A sentinel-2 satellite based analysis of vegetation cover changes in the Kas locality, Darfur, Sudan
**Short Title:**	A sentinel-2 satellite based analysis of vegetation cover changes in the Kas locality, Darfur, Sudan
**Corresponding Author:**	Abdalrahman Ahmed<br>University of Sopron: Soproni Egyetem<br>Sopron, HUNGARY
**Keywords:**	Internally Displaced Persons, Darfur, Armed conflicts, Sentinel-2, Environmental Sustainability
**Abstract:**	Internal displacement of populations due to armed conflicts can substantially impact a region’s Land Use and Land Cover (LULC) and the efforts towards the achievement of Sustainable Development Goals (SDGs). The objective of this study was to determine the effects of conflict-driven Internally Displaced Persons (IDPs) on vegetation cover and environmental sustainability in the Kas locality of Darfur, Sudan.<br>Supervised classification and change analysis were performed on Sentinel-2 satellite images for the years 2016 and 2022 using QGIS software. Five land cover types were successfully classified (agricultural land, vegetation cover, built-up area, sand, and bareland) with overall accuracies of more than 86% and Kappa coefficients greater than 0.78. The results revealed a 20.63% (-3.73 km2) decline in vegetation cover area over the six-year study period, equivalent to an average annual loss rate of -3.44% (-0.62 km2) of vegetation cover. In contrast, agricultural land and built-up areas increased by 6.82% (3.75 km2) and 8.39% (0.81 km2) respectively between the two study years.<br>This study highlights the impacts of IDPs on natural resources and land cover patterns in a conflict-affected region. It also offers pertinent data that can support decision-makers in restoring the affected areas and preventing further environmental degradation for sustainability.
**Order of Authors:**	Abdalrahman Ahmed
	BRIAN Rotich
	Kornel Czimber
**Additional Information:**	
**Question**	**Response**
**Financial Disclosure**<br><br>Enter a financial disclosure statement that describes the sources of funding for the work included in this submission. Review the submission guidelines for detailed requirements. View published research articles from *PLOS ONE* for specific examples.	The author(s) received no specific funding for this work.

Powered by Editorial Manager® and ProduXion Manager® from Aries Systems Corporation

This statement is required for submission and **will appear in the published article** if the submission is accepted. Please make sure it is accurate.<br><br>**Funded studies**<br>Enter a statement with the following details:<br>• Initials of the authors who received each award<br>• Grant numbers awarded to each author<br>• The full name of each funder<br>• URL of each funder website<br>• Did the sponsors or funders play any role in the study design, data collection and analysis, decision to publish, or preparation of the manuscript?<br><br>Did you receive funding for this work?	
**Competing Interests**<br><br>Use the instructions below to enter a competing interest statement for this submission. On behalf of all authors, disclose any competing interests that could be perceived to bias this work—acknowledging all financial support and any other relevant financial or non-financial competing interests.<br><br>This statement is required for submission and **will appear in the published article** if the submission is accepted. Please make sure it is accurate and that any funding sources listed in your Funding Information later in the submission form are also declared in your Financial Disclosure statement.<br><br>View published research articles from *PLOS ONE* for specific examples.	The authors have declared that no competing interests exist

**NO authors have competing interests**<br><br>Enter: *The authors have declared that no competing interests exist.*<br><br>**Authors with competing interests**<br><br>Enter competing interest details beginning with this statement:<br><br>*I have read the journal's policy and the authors of this manuscript have the following competing interests: [insert competing interests here]*<br><br>* typeset	
**Ethics Statement**<br><br>Enter an ethics statement for this submission. This statement is required if the study involved:<br><br>• Human participants<br>• Human specimens or tissue<br>• Vertebrate animals or cephalopods<br>• Vertebrate embryos or tissues<br>• Field research<br><br>Write "N/A" if the submission does not require an ethics statement.<br><br>General guidance is provided below. Consult the submission guidelines for detailed instructions. **Make sure that all information entered here is included in the Methods section of the manuscript.**	N/A

Powered by Editorial Manager® and ProduXion Manager® from Aries Systems Corporation

**Format for specific study types**<br><br>**Human Subject Research (involving human participants and/or tissue)**<br>• Give the name of the institutional review board or ethics committee that approved the study<br>• Include the approval number and/or a statement indicating approval of this research<br>• Indicate the form of consent obtained (written/oral) or the reason that consent was not obtained (e.g. the data were analyzed anonymously)<br><br>**Animal Research (involving vertebrate animals, embryos or tissues)**<br>• Provide the name of the Institutional Animal Care and Use Committee (IACUC) or other relevant ethics board that reviewed the study protocol, and indicate whether they approved this research or granted a formal waiver of ethical approval<br>• Include an approval number if one was obtained<br>• If the study involved *non-human primates*, add *additional details* about animal welfare and steps taken to ameliorate suffering<br>• If anesthesia, euthanasia, or any kind of animal sacrifice is part of the study, include briefly which substances and/or methods were applied<br><br>**Field Research**<br><br>Include the following details if this study involves the collection of plant, animal, or other materials from a natural setting:<br>• Field permit number<br>• Name of the institution or relevant body that granted permission	
**Data Availability**<br><br>Authors are required to make all data underlying the findings described fully available, without restriction, and from the time of publication. PLOS allows rare exceptions to address legal and ethical concerns. See the PLOS Data Policy and FAQ for detailed information.	Yes - all data are fully available without restriction

A Data Availability Statement describing where the data can be found is required at submission. Your answers to this question constitute the Data Availability Statement and **will be published in the article**, if accepted.<br><br>**Important:** Stating 'data available on request from the author' is not sufficient. If your data are only available upon request, select 'No' for the first question and explain your exceptional situation in the text box.<br><br>Do the authors confirm that all data underlying the findings described in their manuscript are fully available without restriction?	
**Describe where the data may be found in full sentences. If you are copying our sample text, replace any instances of XXX with the appropriate details.**<br><br>• If the data are **held or will be held in a public repository**, include URLs, accession numbers or DOIs. If this information will only be available after acceptance, indicate this by ticking the box below. For example: *All XXX files are available from the XXX database (accession number(s) XXX, XXX.).*<br>• If the data are all contained **within the manuscript and/or Supporting Information files**, enter the following: *All relevant data are within the manuscript and its Supporting Information files.*<br>• If neither of these applies but you are able to provide **details of access elsewhere**, with or without limitations, please do so. For example:<br><br>*Data cannot be shared publicly because of [XXX]. Data are available from the XXX Institutional Data Access / Ethics Committee (contact via XXX) for researchers who meet the criteria for access to confidential data.*<br><br>*The data underlying the results presented in the study are available from (include the name of the third party*	All relevant data are within the manuscript and its Supporting Information files.

and contact information or URL).<br>• This text is appropriate if the data are owned by a third party and authors do not have permission to share the data.<br><br>* typeset	
Additional data availability information:	

Manuscript

Click here to access/download;Manuscript;Darfur_Draft_2024.docx

Assessment of the environmental impacts of conflict-driven Internally Displaced Persons: A sentinel-2 satellite based analysis of vegetation cover changes in the Kas locality, Darfur, Sudan

Abdalrahman Ahmed[a, b*], Brian Rotich[c,d] , Kornel Czimber[#a]

a. Institute of Geomatics and Civil Engineering, Faculty of Forestry, University of Sopron, Sopron, Hungary
b. Department of Forest and Environment, Faculty of Forest Science and Technology, University of Gezira, Sudan
c. Institute of Environmental Sciences, Hungarian University of Agriculture and Life Science, Gödöllő, Hungary
d. Faculty of Environmental Studies and Resources Development, Chuka University, Chuka, Kenya

[#a] Current address: Institute of Geomatics and Civil Engineering, Faculty of Forestry, University of Sopron, Sopron, Hungary

Correspondence*:

Email: ix3mzk@uni-sopron.hu (A.A)

Abstract

Internal displacement of populations due to armed conflicts can substantially impact a region's Land Use and Land Cover (LULC) and the efforts towards the achievement of Sustainable Development Goals (SDGs). The objective of this study was to determine the effects of conflict-driven Internally Displaced Persons (IDPs) on vegetation cover and environmental sustainability in the Kas locality of Darfur, Sudan.

Supervised classification and change analysis were performed on Sentinel-2 satellite images for the years 2016 and 2022 using QGIS software. Five land cover types were successfully classified (agricultural land, vegetation cover, built-up area, sand, and bareland) with overall accuracies of more than 86% and Kappa coefficients greater than 0.78. The results revealed a 20.63% (-3.73 km^2) decline in vegetation cover area over the six-year study period, equivalent to an average annual loss rate of -3.44% (-0.62 km^2) of vegetation cover. In contrast, agricultural land and built-up areas increased by 6.82% (3.75 km^2) and 8.39% (0.81 km^2) respectively between the two study years.

This study highlights the impacts of IDPs on natural resources and land cover patterns in a conflict-affected region. It also offers pertinent data that can support decision-makers in restoring the affected areas and preventing further environmental degradation for sustainability.

Introduction

Land use and land cover (LULC) changes are increasingly being viewed as the primary driver of global environmental changes, including greenhouse gas emissions, global climate change, biodiversity, and ecosystem services loss, soil resource loss, and livelihood loss [1–5]. LULC changes are caused by either natural forces, anthropogenic drivers, or both. Changes in LULC occur primarily due to human pressures on natural landscapes at various geographical and temporal scales as influenced by multiple factors that differ by region [6–8]. Natural causes of LULC changes comprise droughts, natural fires, and general changes and variations in climatic

conditions while the common anthropogenic drivers of LULC changes include agricultural expansion, deforestation, urbanization, mining, and armed conflicts [9–12].

Armed conflicts are an extreme form of socioeconomic shocks that can shape future land-use trajectories [13]. These conflicts can have substantial effects on LULC, especially in the conflict zones, and in areas where the displaced population settles [13]. Globally, armed conflicts have resulted in severe demographic, social, economic, and political implications on the lives and livelihoods of the affected populations. At the landscape level, armed conflicts can either trigger increased or reduced pressure on land-based resources, especially vegetation and wildlife [12,14,15]. Warfare can benefit natural systems due to reduced human pressure on the natural environment in the conflict region thereby reducing contamination and environmental degradation [12,16]. On the other hand, the induction of displaced populations from conflict zones into new landscapes can result in the overexploitation of natural resources leading to deforestation, water pollution, agricultural expansion, and land fragmentation [17–20].

Monitoring and mapping changes in LULC is pivotal for environmental management [12]. Satellite Remote Sensing (RS) data integrated with Geographical Information System (GIS) is an important tool for mapping the extent and spatial distribution of forest and land uses based on a stable classification system for change and analysis with the integration of field data [21,22]. It is an adequate tool for monitoring and detecting changes in vegetation cover using multi-temporal data. Images from previous years can be compared to recent years to measure the differences in the sizes and extent of forest cover [21,23]. The use of RS technology is therefore considered a suitable approach for assessing historical and future LULC changes [23]. The advancement in satellite RS technology has revolutionized the approaches to monitoring the Earth's surface [24]. Since its launch in 2015, there has been a high adoption and application of Sentinel-2 images in LULC studies. This can be attributed to its free access policy, high spatial resolution (10 m), high temporal resolution (5 days), and the availability of red-edge bands with multiple applications [24,25]. Sentinel-2 data can also integrate with other remotely sensed data, as part of data analysis, which improves the overall accuracy when working with Sentinel-2 images. In addition, when used with machine-learning classifiers such as Random Forest (RF) and support vector machine (SVM), Sentinel-2 data produces high accuracies (>80%) [24].

The semi-arid Darfur region of Sudan has experienced a protracted humanitarian crisis, characterized by armed conflict, widespread displacement, and environmental degradation since the early 2000s [26,27]. A new wave of violence and insecurity emanating from fighting involving armed movements, government forces, and

armed tribal militia has rocked Darfur since 2014. This conflict has created additional humanitarian crisis in the region as it has led to the displacement of about 321,929 people [27]. The conflict in Darfur has greatly accelerated the processes of environmental degradation that have undermined subsistence livelihoods and the environmental conditions in the area over the recent decades. The emergence of numerous internally displaced persons (IDPs) communities around the major towns of the Darfur region during the conflict has led to the unsustainable utilization of natural resources significantly impacting on the environment through agricultural expansion, overgrazing, and deforestation leading to environmental degradation [28,29]. This environmental degradation has not only exacerbated the vulnerability of IDPs but also contributed to broader ecological and climatic concerns in the region thereby curtailing the efforts in achieving the Sustainable Development Goals (SDGs) [26,30]. Despite these impacts, little research has been done to quantify and map the vegetation cover changes in the region because of IDPs settlement since the conflict resurgence in the year 2014. This study aims to address the urgent problem of understanding the repercussions of conflict-driven IDPs on vegetation cover and overall environmental sustainability in the Kas locality of Darfur, Sudan from 2016 to 2022 using sentinel 2 satellite data. The findings of this study are crucial for informing decision-makers and guiding efforts to restore affected areas, mitigate further environmental degradation, and contribute to the broader objective of achieving Sustainable Development Goals (SDGs) in conflict-affected regions

Methods

Study area

The study area is located between latitudes 12°29´ and 12°32´north and longitudes 24°14´and 24°20´east, covering an estimated area of about 110.21 km^2 (Fig 1). It lies at an altitude of 400 meters above sea level [31]. It is situated in Kas, South Darfur state, around 86 kilometers northwest of the state's capital Nyala [32]. The Kas location has an average temperature of 26°C and average annual rainfall is 519 mm, which varies from north to south (Fig 2). The total population of the locality is estimated at 365,000 people, 76,843 of whom are IDPs [33]. The two major natural resource-based livelihood systems in Darfur are rain-fed agriculture and pastoralism [34]. The Acacia species dominate the forested areas, for instance the Kas Forest reserve. Darfur's labor market continues to be heavily reliant on resources from the forest and rangelands, placing an undue strain on such resources [34].

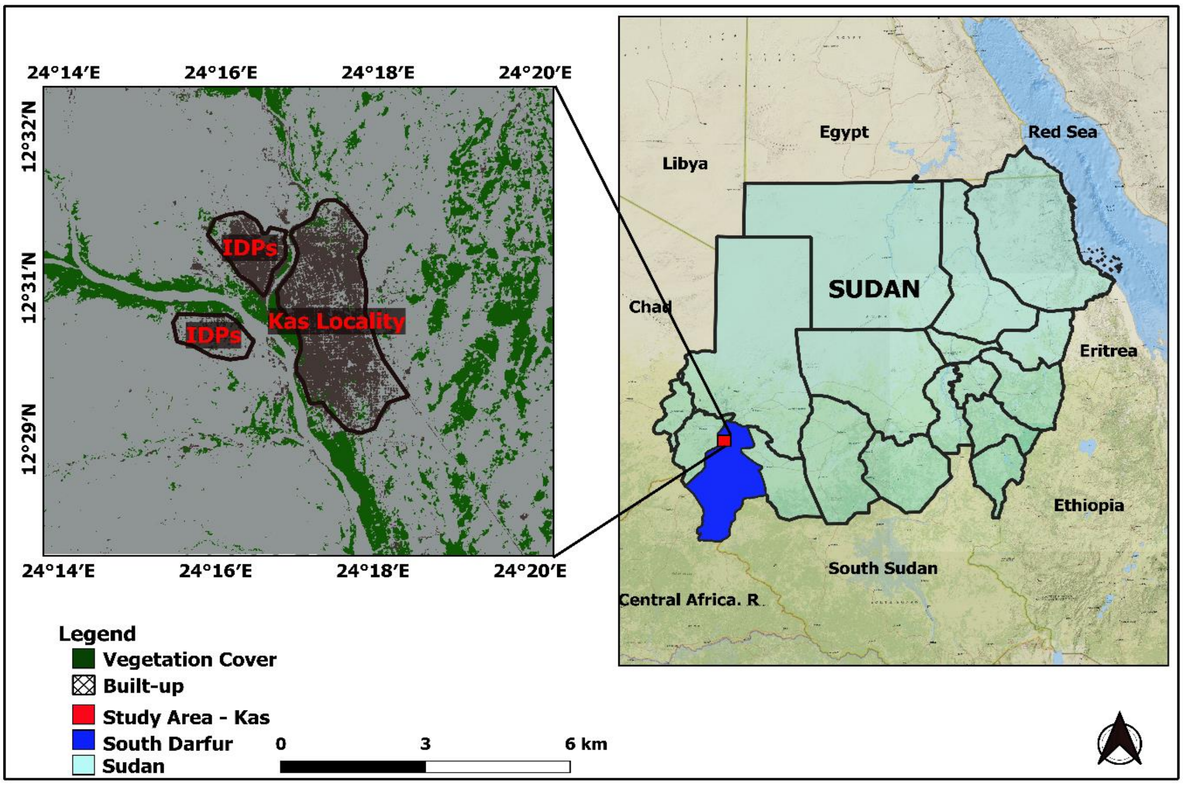

Fig 1. Map of the study area

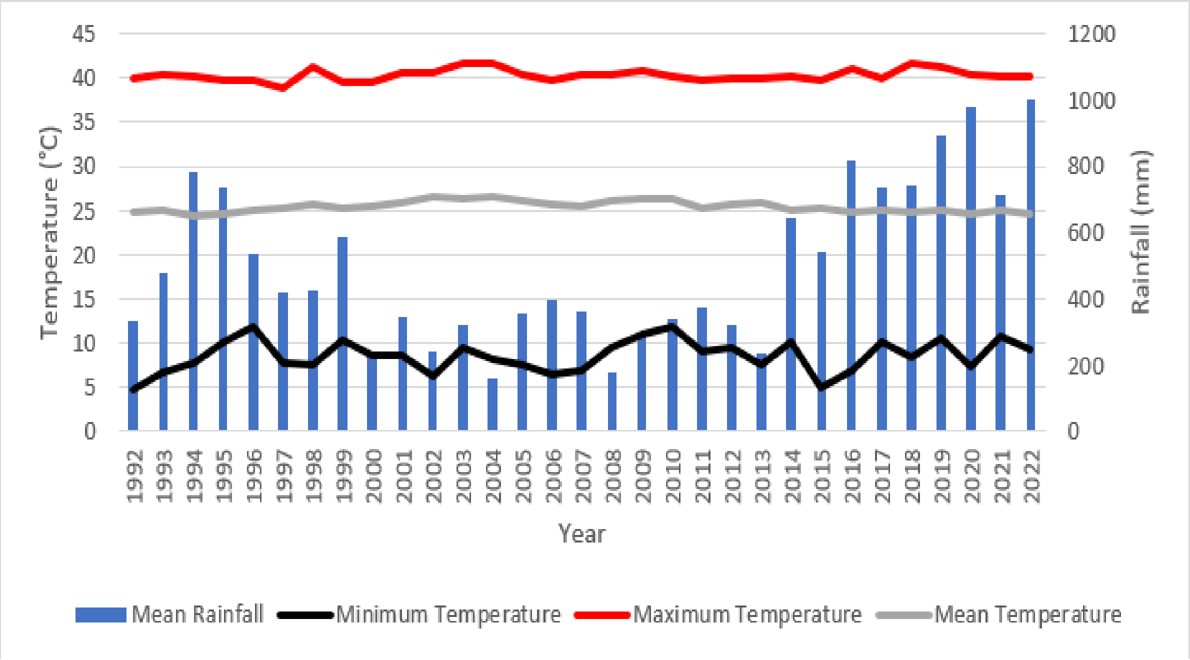

Fig 2. Mean annual rainfall and temperature time series data of the study area from 1992-2022 [50].

Data collection and analysis

Sentinel-2 satellite images with a spatial resolution of 10 meters B2 (Blue), B3 (Green), B4 (Red), and B8 (NIR) were used in this study. These images were downloaded from the United States Geological Survey (USGS) website (http://earthexplorer.usgs.gov/) at Path 178 and Row 52 (Table 1). The dates of the images were chosen to be in the same month and the cloud cover was less than 1%. Rainfall and temperature data were sourced from power data access from the National Aeronautics and Space Administration (NASA) website (https://power.larc.nasa.gov/data-access-viewer/).

Table 1. Details of Sentinel-2 satellite image used in this study

Sensor	Acquisition Date	Path/Row	Grid cell size (Meters)	Bands
**Sentinel 2**	08/12/2016	178/52	10	2,3,4,8
**Sentinel 2**	08/12/2022	178/52	10	2,3,4,8

Image processing

Sentinel-2 Satellite images were selected for this study because of their high resolution (10 meters). The two downloaded satellite data were level-1 products, and the available Sentinel-2 Satellite images of 8th of December 2016 and 2022 were used in this study (Fig 3). Image processing techniques included atmospheric correction, image classification, change detection, and accuracy assessment of the classified images were successfully achieved. All processing steps were carried out in QGIS 3.22 software. Microsoft Office Excel 2013 was used to compute the land cover changes to show the percentages and change rates.

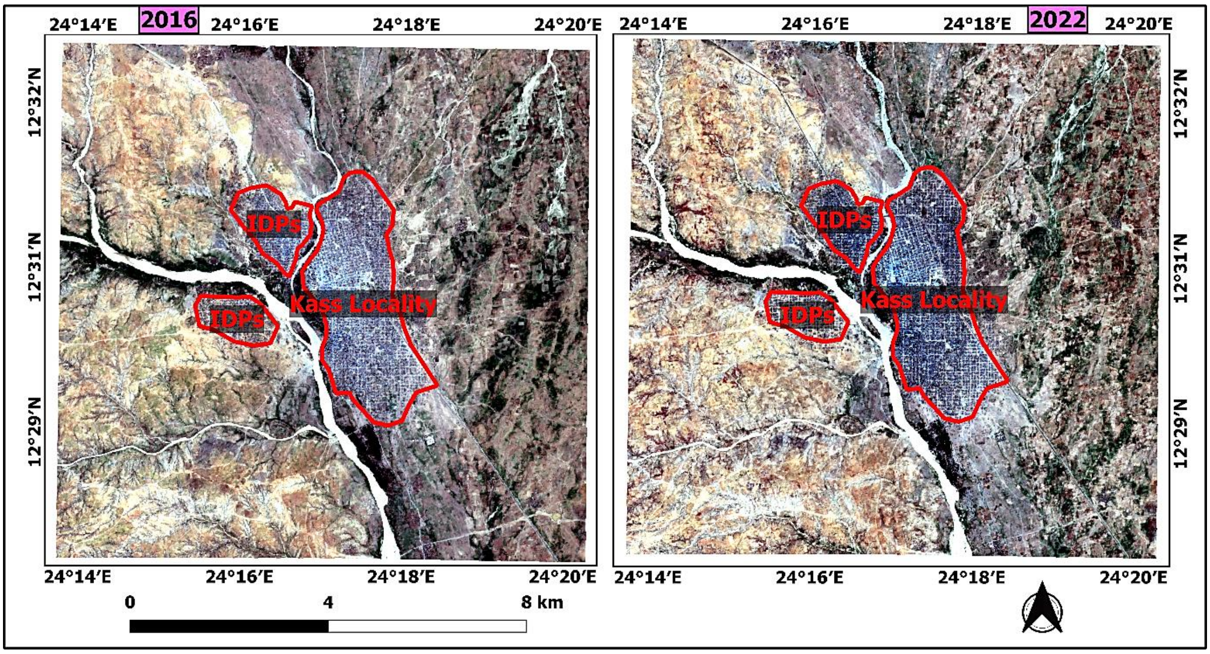

Fig 3. Sentinel 2 Satellite bands combinations (B4, B3, and B2) for the year 2016 and 2022

Image classification and analysis

Supervised classification was performed on Sentinel-2 satellite images acquired for the years 2016 and 2022. The supervised classification was done using Random Forest (RF) classifier in QGIS using the dzetsaka plugin. Manual digitization of vegetation cover, built-up, sand, agricultural land and bareland areas was performed based on high spatial resolution satellite imagery available through the Google Earth Pro software using the historical imagery slider to move between the acquisition dates 2016 and 2022, additionally we used different Sentinel-2 Bands colour composite. The onscreen digitization approach has been widely used and reported in previous studies for obtaining LULC classes, and it has been found to be reliable and accurate [35,36]. We generated 106 samples for the year 2016 and 118 samples for the year 2022, representing the five LULC classes. Then we extracted the corresponding input pixels for both training and testing samples. The training samples were determined using a user defined number of pixels based on the proportional of the polygons per classes.

The images were successfully classified into five classes namely, vegetation cover, bareland, built-up, agricultural land and sand (Table 2, Fig 4). The Sentinel 2 satellite data used in this study were analyzed using QGIS version 3.22, software in combination with Microsoft Office Excel 2016, which was used in computing the LULC changes to show the percentages and change rates in the study area. A classification report containing the area of each class was then generated from the Semi-automatic Classification Plugin (SCP) in QGIS.

Table 2. Description of LULC classes

LULC Class	Description
**Vegetation cover**	Land covered by forests and shrubs. Forests comprise areas >1.5ha with more than 10% of tree cover, with a height exceeding 2 meters. Common tree species include A*cacia spp*, *Balanites aegyptiaca*, and *Eucalyptus spp.* Shrubs are small trees, with heights less than 2 meters, mainly acacia species such as *acacia mellifera*.
**Agricultural land**	Land with less than 10% of scattered vegetated cover, usually cultivated for crop production by the IDPs and the host community during the rainy season (June-October).
**Built-up**	Areas covered by temporary structures, semi-permanent buildings, and permanent buildings. Tents and sheds are examples of temporary structures while semi-permanent buildings comprise huts established for IDPs settlement. Permanent buildings are mainly occupied by the host community.
**Bareland**	Areas without vegetation covered by exposed soils, rocks, rough roads, or degraded lands
**Sand**	Lands dominated by sand with no vegetation cover and no agricultural activities. It includes seasonal water streams (wadies).

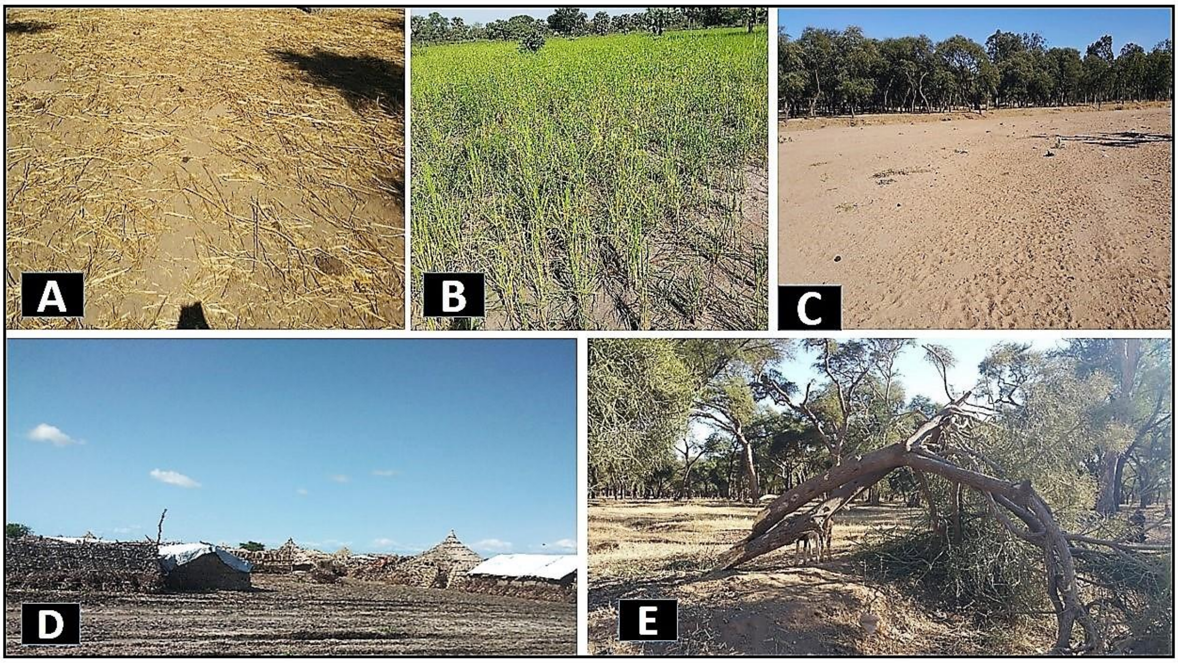

Fig 4. Photos showing the different land cover classes; (A) Bareland (B) Agricultural land (C) Sand with vegetation in the background (D) Built-up (E) Vegetation Cover

Accuracy Assessment

Accuracy assessment is one of the most important steps in the classification process. The aim of accuracy assessment is to quantitatively assess how effectively the pixels were sampled into the correct land cover classes. A total of 12010 pixels were collected for 2016, and 10208 pixels for 2022, then random stratified sample of an equal number of pixels (50%-50%) was applied in each land cover class and the confusion matrix was generated. Error matrix is the most common way to present the accuracy of classified images [37]. Overall, users' and producers' accuracies, and Kappa values were generated from the error matrices. The user's accuracy is calculated by dividing the total number of classified points that agree with the reference data by the total number of classified points for that class. The producer's accuracy is calculated by dividing the total number of classified points that agree with reference data by the total number of reference points for that class. The Kappa value incorporates the off-diagonal elements of the error matrices and represents agreement acquired after removing the proportion of agreement that could be expected to occur by chance. Kappa statistic of the agreement provides an overall assessment of the accuracy of the classification. Kappa coefficient values range from 0 to 1, where 1 infers a perfect agreement (100%) and a value close to zero means that the agreement is not perfect [38]. The data processing workflow for this study is summarized in Fig. 5.

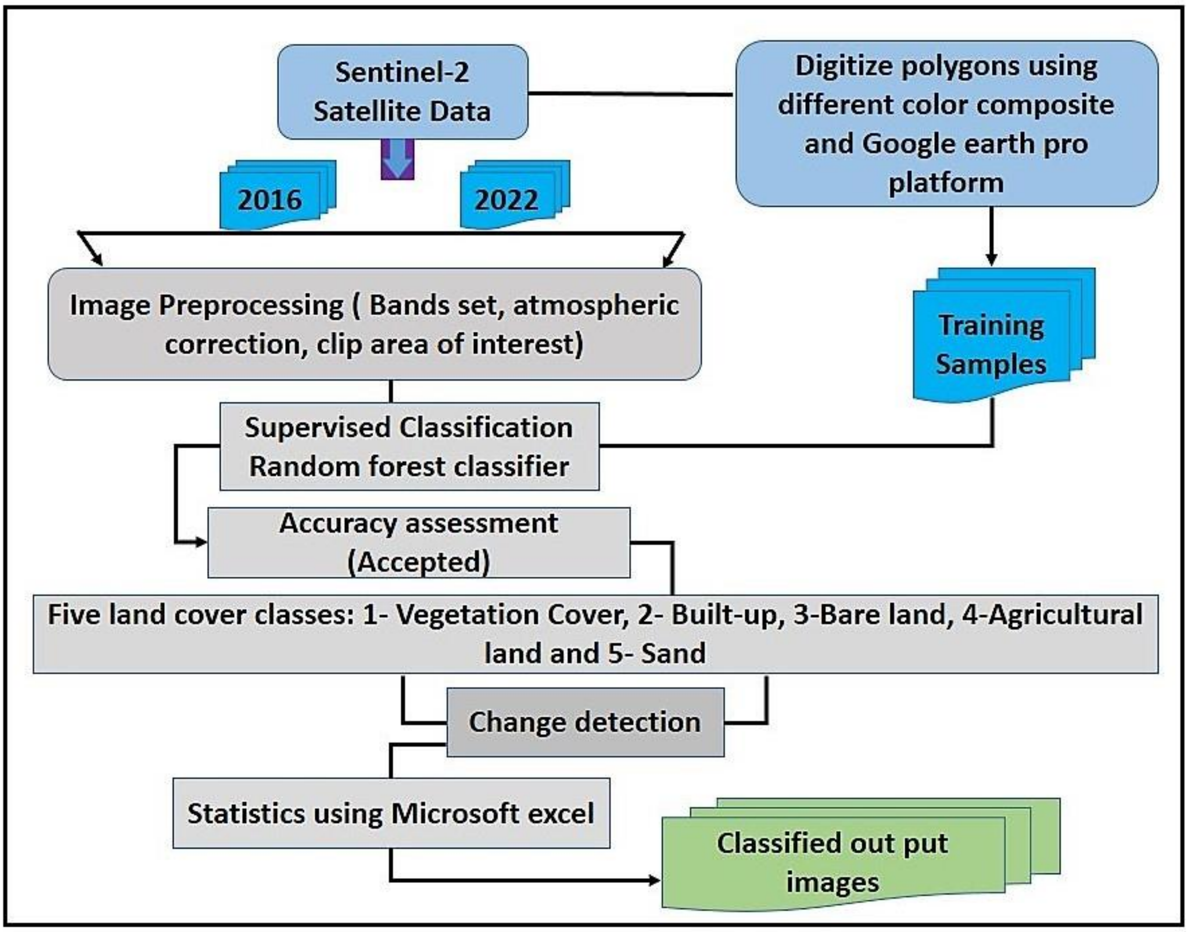

Fig 5. Workflow used to analyze land use and land cover changes

Results

LULC classification accuracy

The overall accuracy, Kappa coefficient, user, and producer accuracies for the five LULC types were calculated and presented in Table 3. The LULC map from 2016 had an overall classification accuracy of 86.10% with a corresponding Kappa coefficient of 0.78, whereas the LULC map for the year 2022 had an overall accuracy of 92.17% with a Kappa coefficient of 0.88. These results are within the acceptable range hence we proceeded and used the classification outputs for the study [38].

Table 3. Accuracy Assessment matrix of classified satellite imagery

LULC class	2016		2022	
	Producer's accuracy (%)	User's accuracy (%)	Producer's accuracy (%)	User's accuracy (%)
**Bareland**	79.15	50.47	83.88	96.22
**Built-up**	96.64	83.71	98.93	89.09
**Vegetation cover**	99.36	99.62	100	99.84
**Sand**	93.43	99.06	80.29	99.63
**Agricultural land**	82.21	96.65	98.34	92.97
**Overall accuracy (%)**	86.10		92.17	
**Kappa coefficient**	0.78		0.88	

Land use Land cover classification and spatial distribution

The results of Sentinel-2 satellite image classification showed that the total coverage of the study area was 110.21 km^2. Individual class areas and percentages for the years 2016 and 2022 are summarized in **Error! Reference source not found.**. In 2016, agricultural land was the dominant LULC class covering almost half (49.88%) of the study area while bareland was the second largest land cover type at 21.62%. Built-up areas and sand had the least area coverage at 8.76% and 3.33 % respectively. Similarly in 2022, the greatest share of LULC was agricultural land (53.28%) followed by bareland (20.91%), while sand had the least cover (3.29%).

Table 4. LULC classes areas and percentages

LULC class	2016		2022	
	Area (km^2)	Percent (%)	Area (km^2)	Percent (%)
**Vegetation cover**	18.08	16.41	14.35	13.02
**Agricultural land**	54.97	49.88	58.72	53.28
**Bareland**	23.83	21.62	23.05	20.91
**Built-up**	9.65	8.76	10.46	9.49
**Sand**	3.67	3.33	3.63	3.29

**Total**	**110.21**	**100**	**110.21**	**100**

The five LULC classes in Kas's locality for the years 2016 and 2022 were also mapped, and their spatial distribution patterns displayed in Fig 6. Built-up areas are located at the center of the study area while vegetation is mainly found in the eastern part of the Kas locality and along the sandy areas. Agricultural land dominates the western part of the study area.

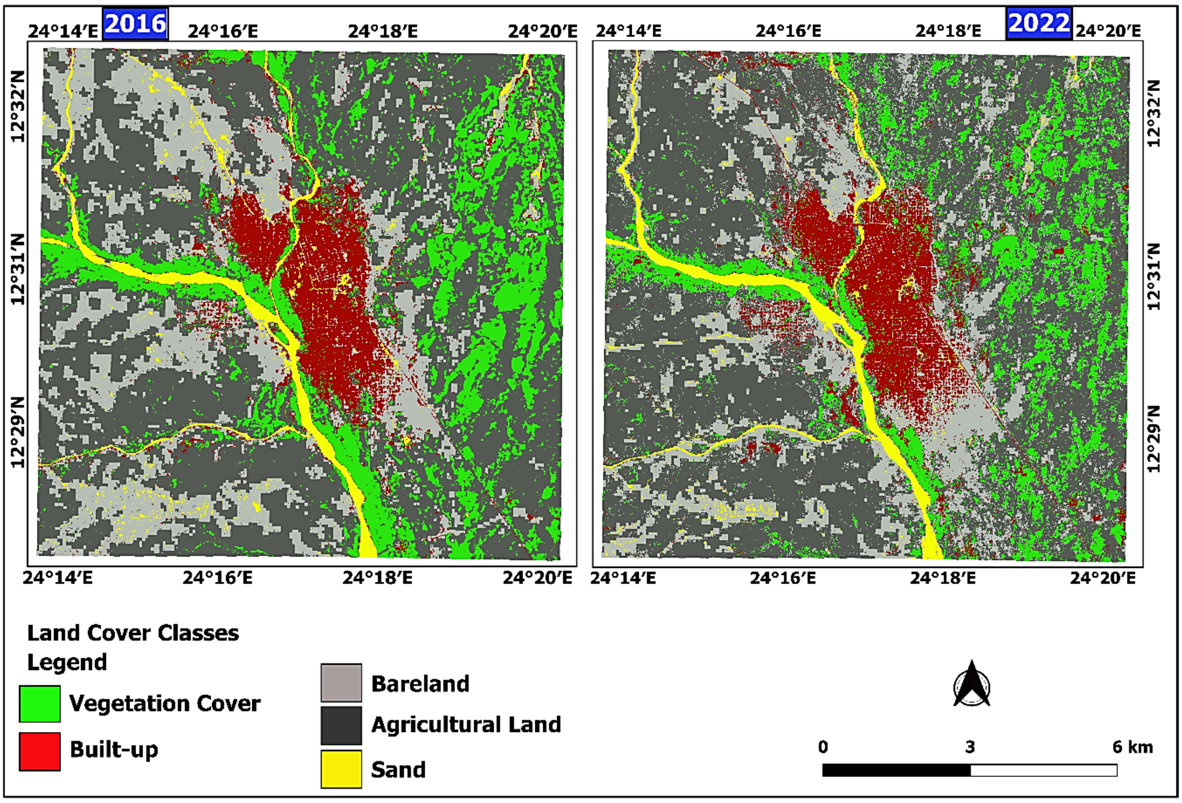

Fig 6. The classified maps of Kas locality for 2016 and 2022

Land use land cover changes

The magnitude and rate of changes in each LULC class between 2016 and 2022 are presented in Table 5. There was an overall reduction in the area under vegetation cover (-3.73 km^2), bareland (-0.78 km^2), and sand (-0.04 km^2). Conversely, agricultural land and built-up areas increased by 3.75 km^2 and 0.81 km^2 respectively. Vegetation cover had the highest mean annual decline rate of -3.44% which translates to a loss of 0.62 km^2 of vegetation cover per annum. Agricultural land gained 0.63 km^2 (1.14%) per year.

Table 5. LULC changes in Kas locality between 2016 and 2022

LULC class	2016 – 2022			
	**Total area change**		**Mean annual change rate**	
	**(km^2)**	**(%)**	**(km^2/year)**	**(%/year)**
**Vegetation cover**	-3.73	-20.63	-0.62	-3.44
**Agricultural land**	3.75	6.82	0.63	1.14
**Bareland**	-0.78	-3.27	-0.13	-0.55
**Built-up**	0.81	8.39	0.13	1.40
**Sand**	-0.04	-1.09	-0.01	-0.18

***Note. (-) = loss, (+) = Gain**

The spatial distribution of vegetation cover changes to other classes and vice versa between 2016 and 2022 is visualized on Fig 7.

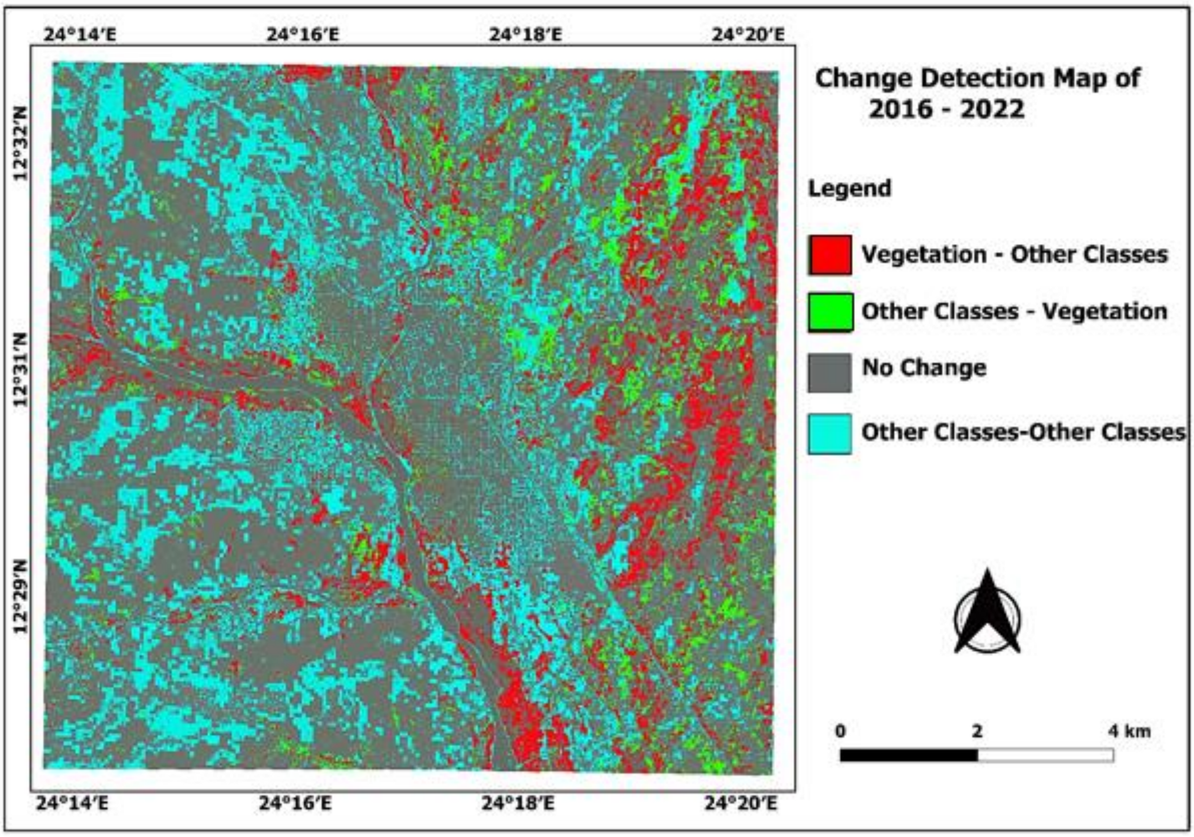

Fig 7. Vegetation cover change detection map of 2016 – 2022

Land cover change transition matrix

For a better understanding of the source and destination of the respective LULC changes, land cover change matrix for the two years of our study was computed (Table 6). LULC changes matrix analysis is important as it shows the direction of change and the LULC type that remains unchanged during the study period [39]. A large proportion of the vegetation cover (6.24 km^2) was converted to agricultural land while about 8.81 km^2 of vegetation remained unchanged between 2016 and 2022. Similarly, significant bareland areas (10.78 km^2) were converted to agricultural land. A minor proportion of the bareland was transformed to vegetation (0.42 km^2) and built-up area (1.11 km^2) over the study period (Table 6).

Table 6. LULC transition matrix (km^2)

		2022					
		**Vegetation cover**	**Agricultural land**	**Bareland**	**Built-up**	**Sand**	**Total**
**2016**	**Vegetation cover**	8.8089	6.244	1.8572	1.07	0.1044	**18.0845**
	**Agricultural land**	4.5167	40.7439	8.0498	1.3595	0.2956	**54.9655**
	**Bareland**	0.4263	10.7824	11.0368	1.1106	0.4736	**23.8297**
	**Built-up**	0.5707	0.7349	1.5101	6.7166	0.1217	**9.654**
	**Sand**	0.0257	0.2162	0.5954	0.1987	2.6376	**3.6736**
	**Total**	**14.3483**	**58.7214**	**23.0493**	**10.4554**	**3.6329**	**110.2073**

Discussion

The LULC classification output of this study were excellent as the overall accuracy were above the recommended threshold of 80% [38,40]. Additionally, the Kappa statistics of the study showed a strong agreement of the classified image since accuracy was within the acceptable range to allow for further LULC changes detection assessment [39]. Five major LULC types (agricultural land, bareland, vegetation cover, sand and built-up) were classified for this study. Agricultural land and bareland were the dominant land cover types, with the former being by far the most dominant land cover type in the study area from 2016 to 2022. There were significant shifts in the areas of the different LULC classes with three classes showing a decline (vegetation cover, bareland, sand) while agricultural land and built-up areas increased. Vegetation was the most reduced land cover type (-

20.63%). The land cover class conversion matrix also revealed that vegetation to non-vegetation conversion increased substantially over the 6-year study period.

The vegetation cover loss in the Kas locality of Darfur can be attributed to resettlement of IDPs from the displaced populations. The estimated populations of the IDPs Kas locality has risen from between 35,000 to 40,000 in 2004 to about 77,843 by the year 2020 [32,41]. Data from the United Nations Office for the Coordination of Humanitarian Affairs [27] further shows that the Darfur region of Sudan has been experiencing newly displaced populations annually from 2003 to 2014 (Table 7).

Table 7. Yearly data of newly displaced population in Darfur [27]

Year	2003	2004	2005	2006	2007	2008	2009	2010	2011	2012	2013	2014
Displaced People	989,920	853,000	No data	270,000	300,000	317,000	175,000	268,000	80,000	114,000	380,000	322,000

The resettlement of IDPs has subsequently increased the human and livestock populations in Kas's locality. The rise in number of IDPs subsequently resulted in extensive agricultural activities to meet their food demands hence the expansion of the agricultural land at the expense of vegetation cover and bareland as is evident in the transition matrix (Table 6). The increased demand for food production often leads to the conversion of the vegetated areas to farms through clear felling of trees to increase the land for crop production. This negatively affects the vegetation cover area while contributing to the expansion of agricultural land [29]. This finding corroborates with that by [13], who reported the establishment of new agricultural areas on Azerbaijani territory, because of refugee migrations due to armed conflicts. A study by [42] further highlights the expansion of agricultural land, resettlement, and population growth as the key drivers of forest fragmentation in the Kaffa Biosphere Reserve of Ethiopia. Most IDPs are pastoralists who migrated with their livestock to the new settlement areas. This leads to increased livestock populations and overgrazing of the shrubs in the vegetated areas. Palatable tree branches are also cut down for fodder, contributing to deforestation. Similar findings were reported by [18] in the Afghan refugee camps in northern Pakistan.

Increase in human population due to conflict related displacements is frequently linked to increased deforestation rates due to overdependence and overutilization of the forest resources [19]. Increased human population in the neighbouring Zalingei IDP camps has previously been associated with considerable decrease in woody vegetation within the camp's vicinity [43]. A study in four hotspot regions around IDP camps in Darfur showed a correlation between decreased vegetation cover with logging of trees as well as removal of grass and shrubs [29]. The vegetation forms a key and readily accessible/available source of energy for the IDPs in the Kas locality in the

form of firewood and charcoal [41]. Unregulated access, however, leads to overexploitation hence a reduction in vegetation cover. These findings conform with that by [14] and [44] who cite overexploitation of forest resources for firewood and charcoal production as a key driver of vegetation cover loss. The forests and shrubs further provide timber, building poles and tree branches which are used in the establishment of the temporary settlement structures by the IDPs (built-up areas) which has increased over time. This exerts additional pressure on the already dwindling vegetation cover in the study area. A study by [14,19] in the Teknaf region of Bangladesh, showed that a mass influx of refugees coupled with a vast expansion of refugee camps led to large scale degradation of forestlands from overharvesting of poles and timber used in building the spontaneous settlements.

There was an increase in the built-up areas (8.39%) of the study area. This can be linked to the the renewed civil conflict which erupted in 2014 emanating from fighting involving armed movements, government forces, and armed tribal militia which led to the displacement of more people hence more IDPs settlement. The built-up areas are likely to further increase with the recent conflicts between the Sudanese Armed Forces, and the paramilitary Rapid Support forces which started in April 2023, as the more people are likely to go back to the IDP camps. The area under sand notably far away from the IDPs camps and settlements slightly decreased by -1.09%, indicating that there has been a minor vegetation regeneration due to minimal disturbance.

Climate change and extreme negative climate variability has previously been reported in Sudan [22]. The variability of climatic conditions in the form of increased temperatures and variability of rainfall in the study area is also likely to play a role in the observed LULC changes [29] . There have been significant variations in the rainfall patterns and temperatures in the study area over the past three decades with an estimated 0.4°C temperature rise per decade [29,43]. Fluctuations in the seasonal precipitation patterns and temperature impacts the natural environment as they have the potential of creating drought-prone conditions [12,43]. Although, climatic variations also influence the changes in the LULC, they play a minor role compared to the direct impacts of IDPs camp-related activities in this region [29,45].

Implications on environmental sustainability

Armed conflict and warfare primarily have a local impact on land systems, but they can also create tele couplings which can negatively affect the achievement SDGs 6, 13, 15 and 16 [46]. The armed conflicts in Sudan have resulted in the displacement of human and livestock populations. This coupled with the drought experienced in the last decade has also led to a massive displacement of the general population. These migrations have brought about overexploitation of forests, water resources, arable land, and pastures. The large numbers of IDPs living in

refugee camps has further resulted in amplified groundwater pollution, air pollution and a rise in respiratory diseases caused by indoor cooking in the camp areas [41]. Additionally, there has been increased conflicts between herdsmen and farmers, worsened by the intervention of militias [47]. An effort by the government of Sudan to mitigate drought and climate change impacts, among others, has focused on the forestry sector with the establishment of new forest plantations [48]. Forests now are more important than ever before for they play a significant role in balancing the earth's carbon dioxide supply and exchange, thereby acting as a key link between the spheres of the earth [49]. Peacebuilding has the potential of boosting vegetation conservation; however, most conservation organizations' core mission in Sudan is not on conflict reduction. Partnerships between conservation and peace building organizations is therefore paramount for the reduction of future drastic land cover changes and the achievement of the aforementioned SDGs [20].

Conclusions

Using Sentinel-2 satellite data and GIS, this study sought to understand how the vegetation cover has changed in South Darfur State between 2016 and 2022 because of IDPs settlement brought about by renewed armed conflicts in year 2014. Study findings show a considerable negative impact of the IDPs on vegetation cover, as seen by the negative change trend. Since the IDPs depend on the forest, shrubs, and scattered trees for their livelihoods, vegetation cover area has experienced a significant reduction due to over exploitation, which affects the natural regeneration process. Other human driven activities like traditional rain-fed agricultural practices and grazing have also led to a reduction in vegetation cover.

These findings underscore the urgent need for targeted interventions and policy responses to address the environmental consequences of conflict-driven displacement. Whereas the consequences armed conflict is typically felt immediately, they have the potential to create long lasting effects on land cover and the achievement of SDGs. Peace efforts can offer long lasting solutions as the IDPs will return to their homes thereby reducing the pressure on the vegetation cover. Implementation of sustainable agricultural management practices and regulated utilization of natural ecosystems like forests and shrubs can help curb vegetation cover loss in the Kas locality. The study offers pertinent data that can help decision-makers plan restoration of the degraded areas and prevent further environmental degradation to promote long-term environmental sustainability.

Acknowledgements

Much gratitude goes to the Sudanese Ministry of Higher Education and Scientific Research for their academic support, as well as the Tempus Public Foundation and the University of Sopron for providing a PhD scholarship to the first author.

Funding

This research did not receive any specific grant from funding agencies in the public, commercial, or not-for-profit sectors.

Declaration of competing interests

The authors declare no conflict of interests

Data availability

Data from this study can be made available by the corresponding author upon reasonable request

Author Contributions

Abdalrahman Ahmed: Conceptualization, Methodology, Formal analysis, visualization, Writing-original draft

Brian Rotich: Writing-original draft, Methodology, Visualization, Writing- Review and editing

Kornel Czimber: Writing-Review and editing, Supervision

References

1. Hailu A, Mammo S, Kidane M. Land Use Policy Dynamics of land use , land cover change trend and its drivers in Jimma Geneti District , Western Ethiopia. Land use policy [Internet]. 2020;99:105011. Available from: https://doi.org/10.1016/j.landusepol.2020.105011

2. Kindu M, Schneider T, Teketay D, Knoke T. Changes of ecosystem service values in response to land use/land cover dynamics in Munessa-Shashemene landscape of the Ethiopian highlands. Sci Total Environ [Internet]. 2016;547:137–47. Available from: http://dx.doi.org/10.1016/j.scitotenv.2015.12.127

3. Lambin EF, Rounsevell MDA, Geist HJ. Are agricultural land-use models able to predict changes in land-use intensity? Agric Ecosyst Environ. 2000;82:321–31.

4. Tesfay F, Tadesse SA, Getahun YS, Lemma E, Gebremedhn AY. Evaluating the impact of land use land cover changes on the values of ecosystem services in the Chacha Watershed, Ethiopia's central highland. Environ Sustain Indic [Internet]. 2023;18:100256. Available from: https://doi.org/10.1016/j.indic.2023.100256

5. Traore M, Lee MS, Rasul A, Balew A. Assessment of land use/land cover changes and their impacts on land surface temperature in Bangui (the capital of Central African Republic). Environ Challenges [Internet]. 2021;4:100114. Available from: https://doi.org/10.1016/j.envc.2021.100114

6. Geist HJ, Lambin EF. Proximate causes and underlying driving forces of tropical deforestation. Bioscience. 2002;52:143–50.

7. Lambin EF, Coomes OT, Turner BL, Geist HJ, Agbola SB, Angelsen A, et al. The causes of land-use and land-cover change : Moving beyond the myths. Glob Environ Chang. 2001;11:261–9.

8. Lambin EF, Geist HJ, Lepers E. Dynamics of land-use and land-cover change in tropical regions. Annu Rev Environ Resour. 2003;28:205–41.

9. Liu Y, Huang X, Yang H, Zhong T. Environmental effects of land-use/cover change caused by urbanization and policies in Southwest China Karst area - A case study of Guiyang. Habitat Int [Internet]. 2014;44:339–48. Available from: http://dx.doi.org/10.1016/j.habitatint.2014.07.009

10. Gbedzi DD, Ofosu EA, Mortey EM, Obiri-Yeboah A, Nyantakyi EK, Siabi EK, et al. Impact of mining on land use land cover change and water quality in the Asutifi North District of Ghana, West Africa. Environ Challenges. 2022;6.

11. Malede DA, Alamirew T, Kosgie JR, Andualem TG. Analysis of land use/land cover change trends over Birr River Watershed, Abbay Basin, Ethiopia. Environ Sustain Indic [Internet]. 2023;17:100222. Available from: https://doi.org/10.1016/j.indic.2022.100222

12. Mohamed MA, Anders J, Schneider C. Monitoring of changes in land use/land cover in Syria from 2010 to 2018 using multitemporal landsat imagery and GIS. Land. 2020;9.

13. Baumann M, Radeloff VC, Avedian V, Kuemmerle T. Land-use change in the Caucasus during and after the Nagorno-Karabakh conflict. Reg Environ Chang [Internet]. 2015;15:1703–16. Available from:

http://dx.doi.org/10.1007/s10113-014-0728-3

14. Hassan MM, Smith AC, Walker K, Rahman MK, Southworth J. Rohingya refugee crisis and forest cover change in Teknaf, Bangladesh. Remote Sens. 2018;10:1–20.

15. Sánchez-Cuervo AM, Aide TM. Consequences of the Armed Conflict, Forced Human Displacement, and Land Abandonment on Forest Cover Change in Colombia: A Multi-scaled Analysis. Ecosystems. 2013;16:1052–70.

16. Ordway EM. Political shifts and changing forests: Effects of armed conflict on forest conservation in Rwanda. Glob Ecol Conserv [Internet]. 2015;3:448–60. Available from: http://dx.doi.org/10.1016/j.gecco.2015.01.013

17. Gorsevski V, Kasischke E, Dempewolf J, Loboda T, Grossmann F. Analysis of the Impacts of armed conflict on the Eastern Afromontane forest region on the South Sudan - Uganda border using multitemporal Landsat imagery. Remote Sens Environ [Internet]. 2012;118:10–20. Available from: http://dx.doi.org/10.1016/j.rse.2011.10.023

18. Lodhi MA, Echavarria FR, Keithley C. Using remote sensing data to monitor land cover changes near afghan refugee camps in northern pakistan. Geocarto Int. 1998;13:33–9.

19. Hassan MM, Duveneck M, Southworth J. The role of the refugee crises in driving forest cover change and fragmentation in Teknaf, Bangladesh. Ecol Inform [Internet]. 2023;74:101966. Available from: https://doi.org/10.1016/j.ecoinf.2022.101966

20. Butsic V, Baumann M, Shortland A, Walker S, Kuemmerle T. Conservation and conflict in the Democratic Republic of Congo: The impacts of warfare, mining, and protected areas on deforestation. Biol Conserv [Internet]. 2015;191:266–73. Available from: http://dx.doi.org/10.1016/j.biocon.2015.06.037

21. Bekele N, Demissie B. Analyzing and Modeling of Geo Spatial Effect on Radio Wave Propagation System Using Geospatial Technologies. J Geogr Inf Syst. 2017;09:777–95.

22. Siddig K, Stepanyan D, Wiebelt M, Grethe H, Zhu T. Climate change and agriculture in the Sudan: Impact pathways beyond changes in mean rainfall and temperature. Ecol Econ [Internet]. 2020;169:106566. Available from: https://doi.org/10.1016/j.ecolecon.2019.106566

23. Sharma R, Rimal B, Baral H, Nehren U, Paudyal K, Sharma S, et al. Impact of land cover change on

ecosystem services in a tropical forested landscape. Resources. 2019;8:1–13.

24. Phiri D, Simwanda M, Salekin S, Ryirenda VR, Murayama Y, Ranagalage M, et al. remote sensing Sentinel-2 Data for Land Cover / Use Mapping : A Review. Remote Sens. 2019;42:14.

25. Abdi AM. Land cover and land use classification performance of machine learning algorithms in a boreal landscape using Sentinel-2 data. GIScience Remote Sens [Internet]. 2020;57:1–20. Available from: https://doi.org/10.1080/15481603.2019.1650447

26. Waal A de. Darfur and the Failure of the Responsibility to Protect. Int Aff. 2007;83:1039–54.

27. OCHA. Sudan: 2014 Population Displacement in Darfur. 2014; Available from: www.unocha.org

28. UNEP. Destitution, distortion and deforestation:The impact of conflict on the timber and woodfuel trade in Darfur. 2008.

29. Kranz O, Sachs A, Lang S. Assessment of environmental changes induced by internally displaced person (IDP) camps in the Darfur region, Sudan, based on multitemporal MODIS data. Int J Remote Sens. 2015;36:190–210.

30. Hagan J, Rymond-Richmond W. The collective dynamics of racial dehumanization and genocidal victimization in Darfur. Am Sociol Rev. 2008;73:875–902.

31. TEARFUND. Darfur: Relief in a vulnerable environment. 2007.

32. CARE SUDAN. Rapid Needs Assessment Report: South Darfur state locations of Kass, Kailek, kabum. 2004.

33. HAC. Household biogas digester for slums, IDPs and refugees camps: case study of Kass South Darfur, Sudan. 9th ICAT. 2017. p. 234.

34. Young BH, Fitzpatrick M, Marshak A, Radday A. Lessons for Taadoud II : Improving Natural Resource Management. 2019.

35. Hansen MC. High-Resolution Global Maps of. Science (80-). 2013;850:850–4.

36. De Sousa C, Fatoyinbo L, Neigh C, Boucka F, Angoue V, Larsen T. Cloud-computing and machine learning in support of country-level land cover and ecosystem extent mapping in Liberia and Gabon. PLoS One. 2020;15:1–24.

37. Plourde L, Congalton RG. Sampling method and sample placement: How do they affect the accuracy of remotely sensed maps? Photogramm Eng Remote Sensing. 2003;69:289–97.

38. Landis JR, Koch GG. The Measurement of Observer Agreement for Categorical Data Data for Categorical of Observer Agreement The Measurement. Biometrics [Internet]. 1977;33:159–74. Available from: http://www.jstor.org/stable/2529310 .

39. Abebe G, Getachew D, Ewunetu A. Analysing land use/land cover changes and its dynamics using remote sensing and GIS in Gubalafito district, Northeastern Ethiopia. SN Appl Sci [Internet]. 2022;4. Available from: https://doi.org/10.1007/s42452-021-04915-8

40. Kindu M, Schneider T, Teketay D, Knoke T. Land Use / Land Cover Change Analysis Using Object-Based Classification Approach in Munessa-Shashemene Landscape of. Remote Sens. 2013;2411–35.

41. Adam W, Ronteltap M, Wolter D, Azrag E. Household biogas digester for slums, IDPs and refugees camps: case study of Kass South Darfur, Sudan. 2020; Available from: https://www.researchgate.net/publication/360929194

42. Mengist W, Soromessa T, Feyisa GL. Forest fragmentation in a forest Biosphere Reserve: Implications for the sustainability of natural habitats and forest management policy in Ethiopia. Resour Environ Sustain [Internet]. 2022;8:100058. Available from: https://doi.org/10.1016/j.resenv.2022.100058

43. Spröhnle K, Kranz O, Schoepfer E, Moeller M, Voigt S. Earth observation-based multi-scale impact assessment of internally displaced person (IDP) camps on wood resources in Zalingei, Darfur. Geocarto Int [Internet]. 2016;31:575–95. Available from: http://dx.doi.org/10.1080/10106049.2015.1062053

44. Rotich B, Ojwang D. Trends and drivers of forest cover change in the Cherangany hills forest ecosystem, western Kenya. Glob Ecol Conserv [Internet]. 2021;30:e01755. Available from: https://doi.org/10.1016/j.gecco.2021.e01755

45. Kranz O, Zeug G, Tiede D, Clandillon S, Bruckert D, Kemper T, et al. Monitoring Refugee/IDP camps to Support International Relief Action. Geoinf Disaster Risk Manag - Examples Best Pract. 2010;51–6.

46. Baumann M, Kuemmerle T. The impacts of warfare and armed conflict on land systems. J Land Use Sci [Internet]. 2016;11:672–88. Available from: http://dx.doi.org/10.1080/1747423X.2016.1241317

47. Naoufal N. Peace and environmental education for climate change: challenges and practices in Lebanon. J

Peace Educ. 2014;11:279–96.

48. Brockhaus M, Di Gregorio M, Djoudi H, Moeliono M, Pham TT, Wong GY. The forest frontier in the Global South: Climate change policies and the promise of development and equity. Ambio [Internet]. 2021;50:2238–55. Available from: https://doi.org/10.1007/s13280-021-01602-1

49. Younis AYI, Jonathan BN, Togun AO, Yasin EHE, Omer SO, Hamad GEAI, et al. Assessment of land use and land cover change on Gum Talha (Acacia seyal var. seyal) forest in Bahar Alarab, Sudan. Biodiversitas. 2022;23:4549–60.

50. NASA. Power data acess [Internet]. 2023. Available from: https://power.larc.nasa.gov/data-access-viewer/
