# Supplementary material for: Assessment of the environmental impacts of conflict-driven Internally Displaced Persons: A sentinel-2 satellite based analysis of land use/cover changes in the Kas locality, Darfur, Sudan
Source: PLoS One. 2024 May 30;19(5):e0304034. doi: 10.1371/journal.pone.0304034 (PMC11139307; doi:10.1371/journal.pone.0304034)
Supplement: Attachment — Submitted filename: Response to Reviewer.docx [file pone.0304034.s006.docx]

**RESPONSE TO REVIEWERS**

Dear reviewer,

We greatly appreciate your input on our paper “**Assessment of the environmental impacts of conflict-driven Internally Displaced Persons: A sentinel-2 satellite based analysis of vegetation cover changes in the Kas locality, Darfur, Sudan”**. After thorough consideration, we have incorporated the suggested change, which has expanded the scope of our study as outlined below. The response to reviewer’ has been addressed in green color, while the corresponding changes to the text are highlighted in track changes in the revised version of the manuscript.

Kind regards

Corresponding author

**Reviewer #1**

Reviewer #1: Change the phrase in the title

"A sentinel-2 satellite based analysis of vegetation cover changes"

to

"A sentinel-2 satellite based analysis of land use/cover changes"

Thank you for the suggestion. We have changed the phrase to “A sentinel-2 satellite based analysis of land use/cover changes”. The new title now reads “**Assessment of the environmental impacts of conflict-driven Internally Displaced Persons: A sentinel-2 satellite based analysis of land use/cover changes in the Kas locality, Darfur, Sudan”.**
